# Real-world efficacy and tolerability of fremanezumab in adults with chronic migraine: a 3-month, single-center, prospective, observational study

**DOI:** 10.3389/fneur.2023.1226591

**Published:** 2023-08-10

**Authors:** Christopher Kjaer Cullum, Basit Ali Chaudhry, Thien Phu Do, Faisal Mohammad Amin

**Affiliations:** ^1^Danish Headache Center, Department of Neurology, Rigshospitalet Glostrup, Faculty of Health and Medical Sciences, University of Copenhagen, Copenhagen, Denmark; ^2^Department of Clinical Medicine, University of Copenhagen, Copenhagen, Denmark; ^3^Danish Knowledge Center on Headache Disorders, Rigshospitalet Glostrup, Glostrup, Denmark; ^4^Department of Neurorehabilitation/Traumatic Brain Injury, Rigshospitalet Glostrup, Faculty of Health and Medical Sciences, University of Copenhagen, Copenhagen, Denmark

**Keywords:** headache, migraine with aura, migraine without aura, CGRP, monoclonal CGRP antibodies

## Abstract

**Background:**

Following the promising pre-marketing placebo-controlled randomized clinical trials of fremanezumab, post-marketing studies are necessary to verify efficacy and tolerability in various real-world settings. The present study assessed real-world efficacy and safety of fremanezumab.

**Methods:**

A 3-month, single-center, prospective, observation study of adults with chronic migraine who were treated with monthly subcutaneous injections of 225 mg fremanezumab in Denmark. The primary outcome was defined as proportion of patients who achieved ≥30% reduction in monthly migraine days (MMDs) from baseline to weeks 9–12. Among secondary outcomes were ≥50 and ≥75% responder rates and the proportion of patients reporting adverse events.

**Results:**

A total of 91 patients with chronic migraine were enrolled and received at least one dose of fremanezumab of whom 89 patients (98%) completed the 3-months treatment period. At baseline, the mean (SD) number of monthly headache days was 24.3 ± 5.8 and mean number of MMDs was 18.5 ± 7.4. The number of patients who achieved ≥30% reduction in MMDs from baseline to weeks 9–12 was 58 (65%), while 45 (51%) and 21 (24%) had ≥50 and 75% reduction in MMD, respectively. Twenty-one patients (23%) reported adverse event, in which the most common were constipation (4.4%), fatigue (4.4%) and dizziness (3.3%). No serious adverse events were reported.

**Conclusion:**

In adult chronic migraine patients with previous failure of conventional oral migraine preventives, fremanezumab was found to be effective and well-tolerated.

## Introduction

Migraine is a disabling headache disorder affecting about 15% of the population. It is characterized by recurrent attacks of moderate to severe headaches, which are typically aggravated by routine physical activity and accompanied by nausea, photophobia, and phonophobia (1). Chronic migraine is a subtype of migraine, defined by at least 15 monthly headache days of which at least 8 days are migraine days for more than 3 months (1). Chronic migraine is associated with impaired quality of life, severe disability, and a considerable socio-economic burden (2, 3). Preventive treatment of chronic migraine is a challenging task and includes specific anticonvulsants, antihypertensives, and antidepressants (2, 4). Fremanezumab, a monoclonal antibody (mAb) against the calcitonin gene-related peptide (CGRP) ligand, is one of the four approved anti-CGRP mAb, that has been approved for prevention of chronic migraine (5). Following the phase III, randomized clinical trials of fremanezumab where they found fremanezumab to be an effective and well tolerated migraine prevention treatment (2, 6–9), post-marketing observational studies are necessary to verify both efficacy and tolerability in various real-world setting. The present study evaluates the efficacy and tolerability of fremanezumab in a 3-month, single-center, prospective observational study that includes adults with chronic migraine who have failed at least two oral medications for migraine prevention.

## Methods

### Study overview

This study was a sub-study of the larger parental CARE (CGRP Antibody Registry) study, which is a registry study of adults with chronic migraine who are eligible for preventive treatment with anti-CGRP mAbs in Denmark. Regulatory approvals were obtained through the relevant ethics committee (J-19085557) and the Danish Data Protection Agency prior to study initiation. All patients provided written informed consent, and the study was conducted in accordance with the Declaration of Helsinki.

### Study design and procedures

We conducted a 3-month, single-center, prospective, observational study of fremanezumab for adults with chronic migraine treated with fremanezumab at the outpatient clinic of the Danish Headache Center. Fremanezumab was monthly administered for all patients. Patients scheduled for treatment with fremanezumab were asked to participate in this observational study. If included, data collected by the clinical personal at the routine clinical visits, were then used for this study. For patients to be treated with CGRP-mAbs at the Danish Headache Center it was a requirement by local practice guidelines, that patients provided a 1-month (30 days) run-in headache diary before treatment start. This is done to establish a baseline headache and migraine frequency in order to determine efficacy of the CGRP-mAbs. Day of treatment start was in this study considered as the baseline visit (1st dose). Three months (90 days) after treatment start all patients were scheduled for a routine clinical follow-up visit for treatment evaluation. This was considered as Month 3 visit in this study. Patients received subcutaneous injections of 225-mg of fremanezumab every month (30-days) from baseline to Month 3 (90 days). At baseline visits, clinical personnel instructed patients and supervised first dose administration to ensure correct technique. Subsequent administrations of fremanezumab for the treatment intervals Month 2 (day-31 to day-60) and Month 3 (day-61 to day-90) were done as self-administration by patients.

Clinical personnel instructed patients to fill out a headache diary (paper format) with daily entries. The diary was used to record the occurrence of headache days and migraine days. At the Month 3 study visit, clinical personnel reviewed the diary and registered number of headache days and number of migraine days for Month 3 (day-61 to day-90).

### Study population

Adults (≥18 years old) with chronic migraine in accordance with the International Classification of Headache Disorders, 3rd edition (ICHD-3) (1), were eligible for study inclusion. According to local practice guidelines, eligibility, and reimbursement criteria for treatment with mAbs targeting CGRP-signaling included lack of efficacy and/or tolerability to at least one antihypertensive and one anticonvulsant approved for migraine prevention. Exclusion criteria included medication-overuse headache (MOH) at the time of enrollment (1). In addition, patients who had previously received treatment with other mAbs targeting CGRP-signaling, i.e., eptinezumab, erenumab, galcanezumab, were excluded. Female patients were also excluded if they were planning to become pregnant, were pregnant, or breastfeeding. Patients who were scheduled to initiate treatment with fremanezumab were screened for eligibility from May 2019 to February 2022.

### Outcomes

The primary outcome was defined as the proportion of patients with ≥30% reduction in monthly migraine days (MMD) at Month 3 (day-61 to day-90) compared with baseline. The secondary outcome was defined as the proportion of patients with ≥50% reduction in MMD at Month 3 (day-61 to day-90) compared with baseline. Exploratory efficacy outcomes were: (1) the proportion of patients with ≥75% reduction in MMD at Month 3 (day-61 to day-90) compared with baseline, (2) the proportion of patients with ≥30% reduction in MHD at Month 3 (day-61 to day-90) compared with baseline, (3) the proportion of patients with ≥50% reduction in MHD at Month 3 (day-61 to day-90) compared with baseline, (4) the proportion of patients with ≥75% reduction in MHD at Month 3 (day-61 to day-90) compared with baseline, (5) the absolute change in MMD at Month 3 (day-61 to day-90) compared with baseline, (6) the absolute change in monthly headache days (MHD) at Month 3 (day-61 to day-90) compared with baseline. Tolerability and safety outcomes were: (1) the number of any adverse events during the 3-month treatment period, and (2) the number of any serious adverse events during the 3-month treatment period.

### Statistical analysis

We calculated outcomes based on headache diary entries. We report continuous or categorical variables as means with either 95% confidence interval (95% CI) or standard deviation (SD). We report data with a skewed distribution as medians with interquartile range. MHD and MMD are reported over a 30-day period. Responder rates are reported as proportions of patients who provided data after all 3 months of treatment. We conducted a complete case analysis of efficacy data, which were reported as observed. Tolerability and safety data were reported for all patients who received at least one administration of fremanezumab. Statistical analyses were conducted with R version 4.2.0 for Windows.

## Results

**Figure 1:**
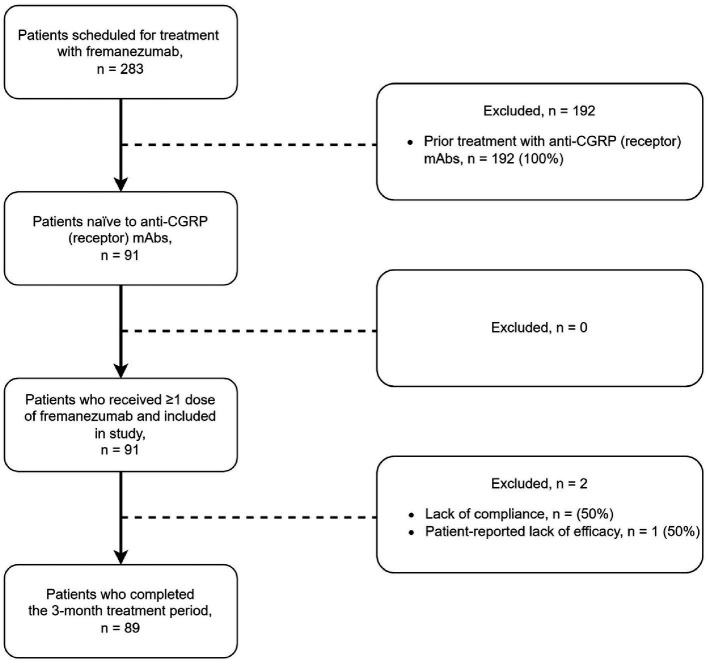
Patient flow. All patients received at least one dose of 225 mg fremanezumab. Evalution occurred at month 3 (day-61 to day-90).

A total of 283 patients were scheduled for treatment with fremanezumab; 192 were excluded due to prior treatment with mAbs targeting CGRP-signaling. Thus, we included 91 patients with chronic migraine, who received at least one dose of fremanezumab between May 2019 to February 2022, were included in the study. Of these, 89 (98%) of 91 patients completed the 3-month treatment period (Figure 1). The mean age of patients was 46.3 (±11.2) years, and 80 (88%) of 91 patients were women. The baseline disease burden was 24.3 (±5.8) MHD and 18.5 (±7.4) MMD. The median number of previous preventive migraine medications was 6 (IQR: 4–8). Thirty-eight (42%) of 91 patients used concomitant preventive migraine medications at the time of the first fremanezumab administration. Baseline characteristics of the study population are summarized in (Table 1).

**Table 1:** Study population baseline characteristics.

Study population characteristics	All patients (*n* = 91)
Age, mean, years (SD)	46.3 (11.2)
Women, *n* (%)	80 (88%)
Number of patients with comorbid psychiatric disorder, *n* (%)	16 (17%)
Number of patients with migraine without aura, *n* (%)	77 (85%)
Number of patients with migraine with aura, *n* (%)	23 (25%)
Number of monthly headache days, mean, days (SD)	24.3 (5.8)
Number of monthly migraine days, mean, days (SD)	18.5 (7.4)
Number of prior failed preventive medications, median (IQR)	6 (4–8)
Number of patients using concomitant preventive medication at baseline, *n* (%)	38 (42%)

*All* patients were diagnosed with chronic migraine without medication overuse headache. Abbreviations: SD, standard deviation; IQR, interquartile range.

### Efficacy

**Figure 2:**
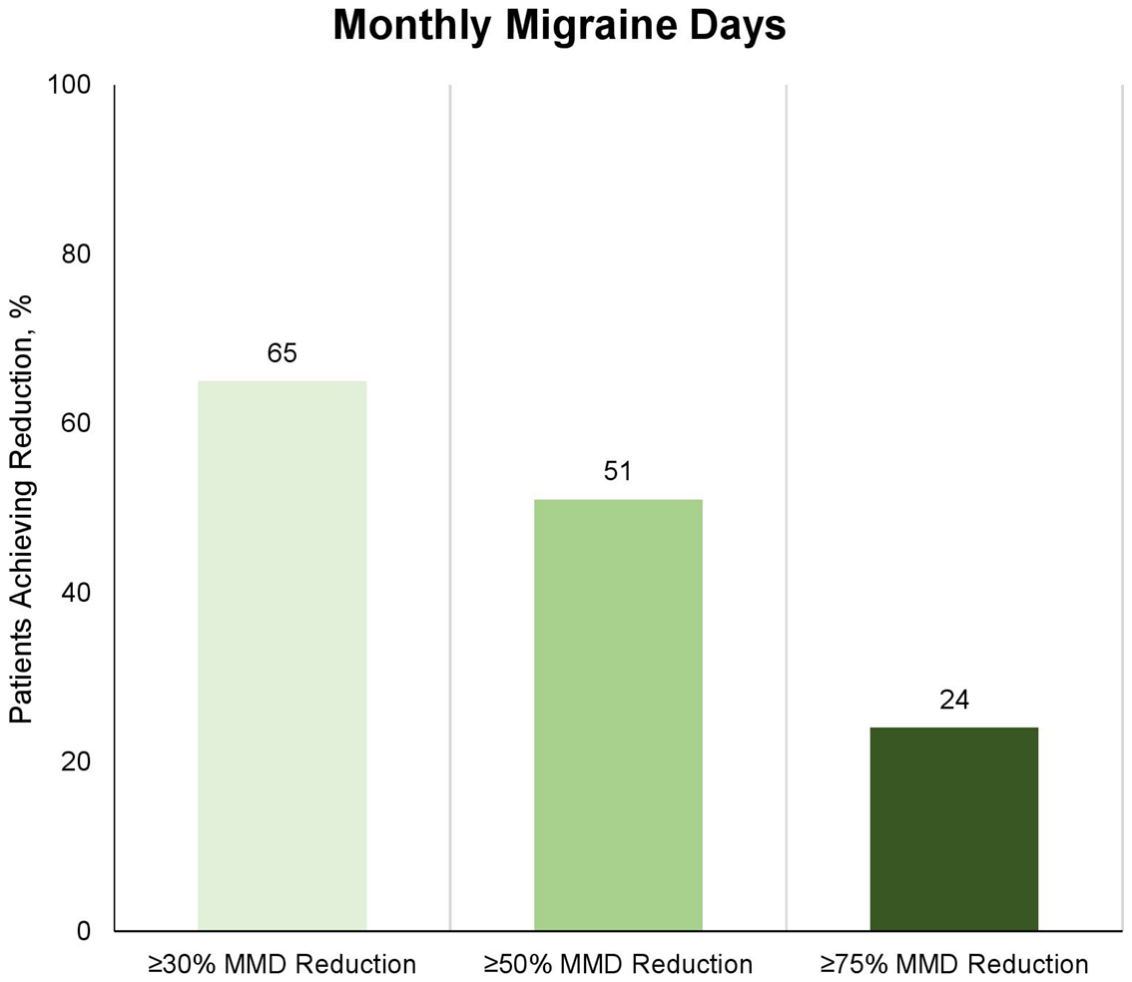
Proportion of patients with ≥30%, ≥50%, and ≥75% reduction in number of monthly migraine days at month 3 (day-61 to day-90) compared to baseline. Eighty-nine of 91 patients completed the 3-month treatment period and provided data for the efficacy analysis. Numbers depicts proportion of patients, who achieved the corresponding reduction in monthly migraine days; light: ≥30%, medium: ≥50%, and dark: ≥75%.

**Figure 3:**
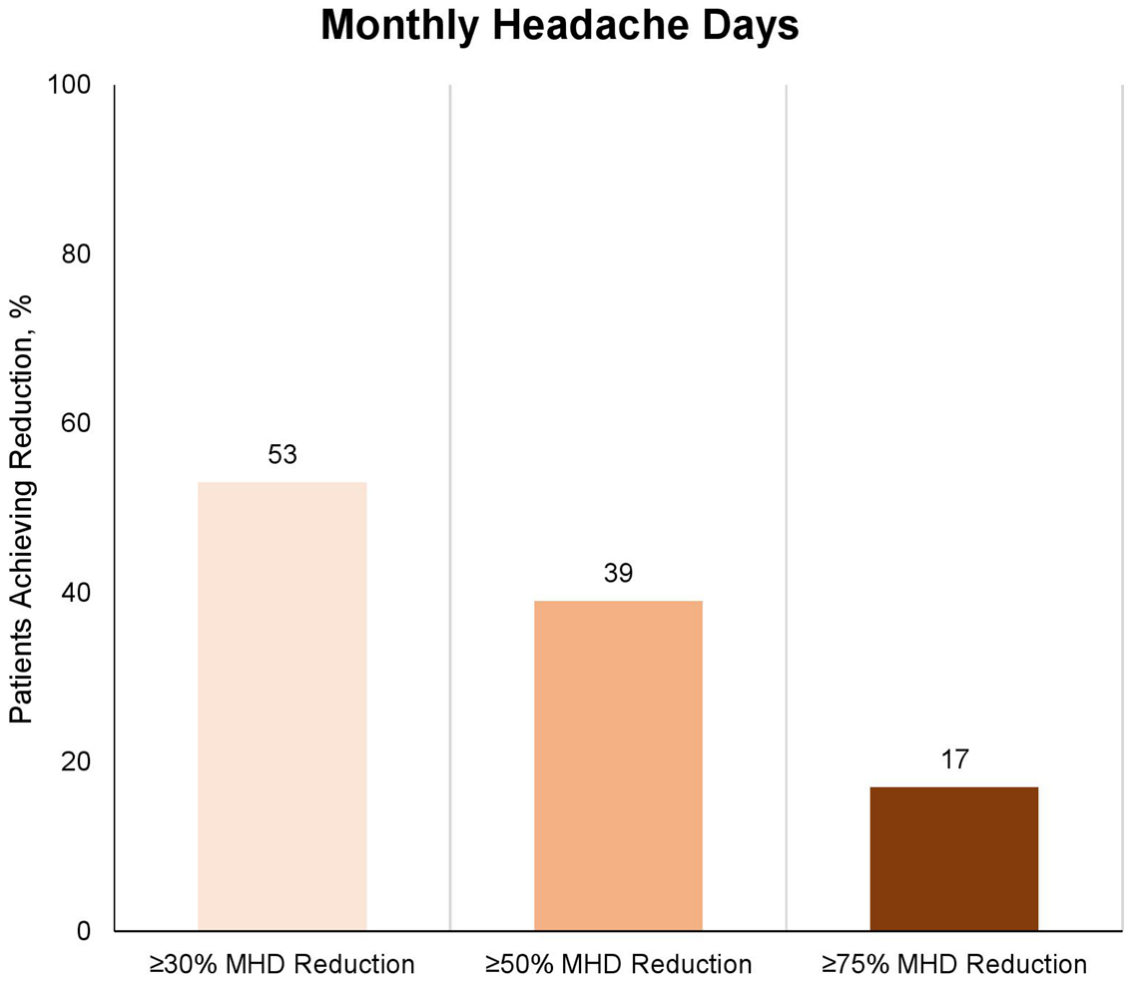
Proportion of patients with ≥30%, ≥50%, and ≥75% reduction in number of monthly headache days at month 3 (day-61 to day-90) compared to baseline. Eighty-nine of 91 patients completed the 3-month treatment period and provided data for the efficacy analysis. Numbers depicts proportion of patients, who achieved the corresponding reduction in monthly migraine days; light: ≥30%, medium: ≥50%, and dark: ≥75%.

Eighty-nine patients provided data for the Month 3 evaluation (day-61 to day-90). The proportion of patients with ≥30% reduction in MMD from baseline to Month 3 (day-61 to day-90) was 58 (65%) of 89 patients (Figure 2). The proportion of patients with ≥50 and 75% reduction in MMD from baseline to Month 3 (day-61 to day-90) was 45 (51%) and 21 (24%) of 89 patients, respectively (Figure 2). The proportion of patients with ≥30%, ≥50 and 75% reduction in MHD from baseline to Month 3 (day-61 to day-90) was 47 (53%), 35 (39%), and 15 (17%) of 89 patients, respectively (Figure 3). The absolute change in MMD from baseline to Month 3 (day-61 to day-90) was −7.3 (95% CI, −9.0 to −5.5) days. The absolute change in MHD from baseline to Month 3 (day-61 to day-90) -8.2 (95% CI, −10.0 to −6.3) days.

### Tolerability and safety

A total of 25 adverse events occurred during the 3-month treatment period. Adverse events were reported by 21 (23%) of 91 patients, who received at least one dose of fremanezumab. The most common adverse events were constipation (*n* = 4), fatigue (*n* = 4), and dizziness (*n* = 3). No patients discontinued treatment during the 3-month period due to adverse events. No serious adverse events occurred. Tolerability and safety data are summarized in (Table 2).

**Table 2:** Treatment-emergent adverse events.

Event	All Patients (*n* = 91)
Number of adverse events, *n*	25
Number of serious adverse events, *n*	0
Number of patients with adverse events, *n* (%)	21 (23%)
Number of patients with serious adverse events, *n* (%)	0 (0%)
Number of patients with adverse events leading to treatment discontinuation, *n* (%)	0 (0%)
*Most frequent any adverse event Constipation, *n* (%) Dizziness, *n* (%) Fatigue, *n* (%) Arthralgia, *n* (%) Injection site reaction, *n* (%) Nausea, *n* (%) Rash, *n* (%) Weight gain, *n* (%)	4 (4%) 4 (4%) 3 (3%) 2 (2%) 2 (2%) 2 (2%) 2 (2%) 2 (2%)

* Adverse events occurring in ≥2% of patients.

## Discussion

This is the first real-world study of fremanezumab for adults with chronic migraine in Denmark. The main findings of the present study were that 65% (58 out of 89) of the patients achieved ≥30% reduction in MMDs, and 51% achieved ≥50% reduction in MMDs. About one-quarter (23%) of the patients reported adverse events. The most common adverse events were constipation and dizziness. There were no serious adverse events.

The criteria for treatment reimbursement for CGRP-mAbs in Denmark (by regulatory authorities) was set to at least 30% response. We therefore set similar cut-off response value of 30% in our study, in contrast to the recommendations of IHS guidelines for clinical trials in chronic migraine (10). The mean reduction in migraine days in our patient group was 7.3 days per month (baseline, 18.5 days per month; Month-3, 11.2 days per month). This was moreover a patient group, who had failed on multiple conventional oral migraine preventives (median 6, IQR 4–8). The fact that 75% of the patients in this trial showed no response to at least four different standard oral migraine preventives may suggest that the migraine patients in question are among the more challenging to treat patients. However, we found a 50% responder rate at 51% in our study, which is comparable to both pre-marketing phase III 41 34% (7), 47.7% (8), and other similar real-world studies (11), that have been published. Efficacy of fremanezumab in patients without failures on several other conventional oral preventive emphasizes a key role of CGRP in migraine pathophysiology. Moreover, the poor effect fremanezumab has on the monthly headache days compared to monthly migraine days in our study, emphasizes that the effect of anti-CGRP mAbs is migraine mechanism-based.

Most recently, a larger pan-European multi-center real-world study of fremanezumab reported ≥50% responder rate at 56.7% (*n* = 224) at Month 3 and 52.6% (*n* = 156) at Month 6 compared to baseline (12). Although, post-marketing real-world data, including the presented, may apparently demonstrate slightly better efficacy of fremanezumab compared to the pre-marketing reports (e.g., 51 vs. 47%, respectively), it must be taken in account that real-world data are not placebo controlled.

Adverse effects were reported by 23% of the patients, in which fatigue (4.4%) and constipation (4.4%) were the most frequent. Overall, there was a low proportion of patients who reported adverse events in our study compared to other phase III studies of similar 3-months treatment periods, where between 45% (7), and 71% (9), of the patients reported adverse events. Study populations are not directly comparable as our study had fewer patients (89) compared to 283, 285 and 379, respectively. In another real-world study of similar treatment period and population size (*N* = 67), 5.7% of the patients reported any adverse event (11). This indicates that fremanezumab is well-tolerated by patients in real-world clinical practice. However, in real-world settings, some adverse events might be under-reported, simply because patients are not asked to actively register every unusual experience during treatment with fremanezumab.

Our study differs methodologically from other post-marketing studies in terms of being a real-world experience study, where prospectively collected data (by clinical personal) in the real-world clinical settings were retrospectively analyzed (by the research team). Moreover, only CGRP mAbs naïve adults with pure chronic migraine and without medication overuse headache were included in the present study. Compared to our study, the 50% responder rate seem to be slightly higher (62.6%; 72.9%) in similar studies (13, 14) where adults with medication overuse headache were also included. Most likely, the better response may be caused by the cessation of medication overuse after initiation of fremanezumab, which questions the necessity to get rid of medication overuse headache before initiation of CGRP mAbs. Unlike most other post-marketing studies (15), we did not include other patient reported outcome measures, as collection of these data does not reflect real-world in our clinic as in many other clinics across the world.

### Study limitations

A major limitation of the present study was headache and migraine days rather than pain intensities were recorded systematically. Moreover, there was a short observational period, which may be insufficient to assess delayed effects of fremanezumab on both efficacy and tolerability. However, in a similar pan-European real-world study, which also included 65 Danish patients, there was no substantial difference in efficacy between month 3 and 6 (12). Another limitation is the lack of use of patient-reported outcomes. Additionally due the nature of real-world studies having no placebo-controlled group, placebo cannot be ruled out as a contributing factor.

## Conclusion

Migraine prevention with fremanezumab was found to be effective and tolerable for difficult to treat chronic migraine patients. Moreover, the efficacy was comparable with the placebo-controlled pre-marketing studies as well as other post-marketing open-label real-world studies.

## Data availability statement

The raw data supporting the conclusions of this article will be made available by the authors, without undue reservation.

## Ethics statement

The studies involving humans were approved by Ethics committee (J-19085557) and the Danish Data Protection Agency. The studies were conducted in accordance with the local legislation and institutional requirements. The participants provided their written informed consent to participate in this study.

## Author contributions

All authors listed have made a substantial, direct, and intellectual contribution to the work and approved it for publication.

## Conflict of interest

TD reports personal fees from Teva, outside of the submitted work. FA has received Honoria or personal fees from Pfizer, Teva, Novartis, Lundbeck and Eli Lilly for lecturing or participating in advisory boards; is principal investigator for phase IV trials sponsored by Novartis and by Teva; serves as president of Danish Headache Society and board member of the European Headache Federation; serves as associate editor for Acta Neurol Scand, Front Neurol, Front Res Pain, and Headache Medicine; serves as junior associate editor for Cephalalgia and Cephalalgia Reports; member of the editorial board of J Headache Pain.

The remaining authors declare that the research was conducted in the absence of any commercial or financial relationships that could be construed as a potential conflict of interest.

## Publisher’s note

All claims expressed in this article are solely those of the authors and do not necessarily represent those of their affiliated organizations, or those of the publisher, the editors and the reviewers. Any product that may be evaluated in this article, or claim that may be made by its manufacturer, is not guaranteed or endorsed by the publisher.

## Abbreviations

CGRP: Calcitonin gene-related peptide
ICHD-3: International Classification of Headache Disorders, 3rd edition
MHD: monthly headache days
MMD: monthly migraine days
MOH: medication-overuse headache
